# QuickStats

**Published:** 2014-11-07

**Authors:** FROM THE NATIONAL CENTER FOR HEALTH STATISTICS

**Figure:**
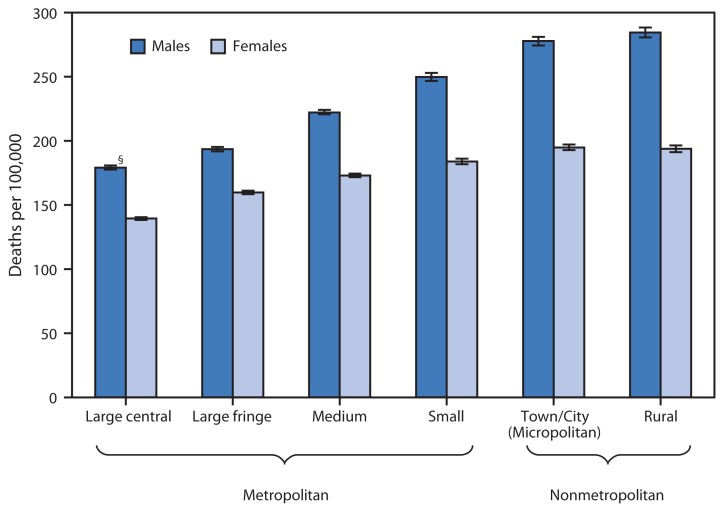
Age-Adjusted Death Rates from Chronic Obstructive Pulmonary Disease (COPD)* Among Persons Aged ≥55 Years, by Sex and Urbanization of County of Residence^†^— United States, 2009–2011 * Per 100,000 standard population. Deaths from COPD are those coded J40–J44 in the *International Classification of Diseases, 10th Revision*. ^†^ Counties were classified into urbanization levels based on a classification scheme that considers metropolitan/nonmetropolitan status, population, and other factors. ^§^ 95% confidence interval.

During 2009–2011, higher death rates for COPD among persons aged ≥55 years were associated with more rural localities, with rates increasing steadily from the least to the most rural county. For males, the age-adjusted COPD death rate in rural counties was 59% higher than in large central metropolitan counties (284.3 versus 178.9 deaths per 100,000 population). For females, the age-adjusted COPD death rate in rural counties was 39% higher than in large central metropolitan counties (193.6 versus 139.3 deaths per 100,000 population). COPD death rates for males were 21% to 47% higher than for females, with the largest differentials observed in nonmetropolitan counties (i.e., town/city and rural counties).

**Sources:** National Vital Statistics System. County-level mortality file. Available at http://www.cdc.gov/nchs/deaths.htm and http://wonder.cdc.gov/mortsql.html.

Ingram DD, Franco SJ. 2013 NCHS urban-rural classification scheme for counties. Vital Health Stat 2014;2(166). Available at http://www.cdc.gov/nchs/data/series/sr_02/sr02_166.pdf.

**Reported by:** Deborah D. Ingram, PhD, ddingram@cdc.gov, 301-458-4733.

